# LncRNA AW112010 Promotes Mitochondrial Biogenesis and Hair Cell Survival: Implications for Age-Related Hearing Loss

**DOI:** 10.1155/2019/6150148

**Published:** 2019-10-27

**Authors:** Zhongwu Su, Hao Xiong, Jiaqi Pang, Hanqing Lin, Lan Lai, Huasong Zhang, Weijian Zhang, Yiqing Zheng

**Affiliations:** ^1^Department of Otolaryngology, Sun Yat-sen Memorial Hospital, Sun Yat-sen University, Guangzhou, China; ^2^Institute of Hearing and Speech-Language Science, Sun Yat-sen University, Guangzhou, China

## Abstract

Long noncoding RNA (lncRNA) disorder has been found in many kinds of age-associated diseases. However, the role of lncRNA in the development of age-related hearing loss (AHL) is still largely unknown. This study sought to uncover AHL-associated lncRNAs and the function. RNA-sequencing was conducted to profile lncRNA expression in the cochlea of an early-onset AHL mouse model. RT-qPCR assay was used to validate the expression pattern of lncRNAs. ATP assay, JC-1 assay, mitochondrial probe staining, CCK-8 assay, Western blot, and immunocytochemistry were performed to detect the effects of lncRNA AW112010 in HEI-OC1 cells and the mouse cochlea. We identified 88 significantly upregulated lncRNAs and 46 significantly downregulated lncRNAs in the cochlea of aged C57BL/6 mice. We focused on the significantly upregulated AW112010. Silencing of AW112010 decreased the ATP level, mitochondrial membrane potential, and cell viability and increased mitochondrial ROS generation under oxidative stress in HEI-OC1 cells. AW112010 overexpression promoted cell survival in HEI-OC1 cells. AW112010 knockdown reduced mitochondrial mass and impaired mitochondrial biogenesis in HEI-OC1 cells. Activation of mitochondrial biogenesis by resveratrol and STR1720 promoted cell survival. The mitochondrial biogenesis process was activated in the cochlea of aged mice. Moreover, AW112010 regulated AMPK signaling in HEI-OC1 cells. Transcription factor Arid5b elevated in the aged cochlea and induced AW112010 expression and mitochondrial biogenesis in HEI-OC1 cells. Taken together, lncRNAs are dysregulated with aging in the cochlea of C57BL/6 mice. The Arid5b/AW112010 signaling was induced in the aged mouse cochlea and positively modulated the mitochondrial biogenesis to maintain mitochondrial function.

## 1. Introduction

Age-related hearing loss (AHL), also known as presbycusis, is the most common sensory disorder in old people, affecting about 20-40% of people by age 65 years and older [1, 2]. It significantly affects the daily communication of the old people. Intrinsic factors (e.g., genetic predisposition) and extrinsic factors (e.g., noise exposure) together result in the occurrence of AHL during aging [1]. The irreversible loss of cochlear hair cells is one of the major pathological changes of AHL [3–5]. Oxidative stress, mitochondrial DNA mutations/deletions, decreased autophagy, and microRNA disorder account for the death of hair cells [1, 2, 4, 5]. However, the mechanism of hair cell loss is still not fully understood.

Long noncoding RNAs (lncRNAs) are a class of RNA that are longer than 200 nucleotides and do not have the potential to code proteins [6, 7]. Recent studies have revealed that lncRNAs play significant roles in the regulation of gene expression and participate in multiple biological processes, including cell growth, apoptosis, and differentiation [6, 7]. LncRNA disorder has been found in many kinds of diseases, such as cancer and cardiovascular and neurodegenerative diseases [8]. LncRNAs have also been reported to be involved in the pathophysiological processes in the ear. A recent study revealed differential lncRNA profile between two developmental stages of the mouse inner ear sensory epithelium of the cochlea and vestibule, suggesting a possible role for lncRNAs in regulating hearing and balance [9]. A study in a Chinese population unveiled that lncRNA HOTAIR polymorphism was associated with the occurrence of noise-induced hearing loss [10]. Nevertheless, it remains largely unknown whether lncRNAs participate in the development of AHL.

Mitochondria have a vital role in maintaining cellular homeostasis [11, 12]. Growing evidence suggests that mitochondrial dysfunction participates in aging diseases, such as diabetes, neurodegenerative disease [12], and AHL [1]. Mitochondrial biogenesis is a tightly regulated process to generate new mitochondria and plays an important role in maintaining normal mitochondrial function [11, 12]. The progress is orchestrated by a series of transcription factors, such as peroxisome proliferator-activated receptor gamma coactivator 1-alpha (PGC-1*α*) and mitochondrial transcription factor A (TFAM), and is under the control of AMP-activated protein kinase (AMPK), the mammalian target of rapamycin (mTOR), and insulin-like signaling (ILS) pathways [11, 12]. As important gene regulation factors, lncRNAs also modulate the expression of genes involved in the mitochondrial biogenesis process [13].

In the present study, we hypothesized that lncRNAs are dysregulated in AHL and play harmful or protective roles in the survival of cochlear hair cells. To address this, we firstly profiled the lncRNA expression in the cochlear tissues of an early-onset AHL mouse model and identified lncRNA AW112010 significantly upregulated in the aged cochlea. Next, we analyzed the effect of AW112010 on mitochondrial function and biogenesis and on the activity of AMPK signaling. Finally, the upstream regulator of AW112010 was explored.

## 2. Materials and Methods

### 2.1. Animals

50 C57BL/6 mice (Laboratory Animal Center, Sun Yat-sen University, Guangzhou, China) were divided into 2 groups: a “young” group (4 weeks old, 25 subjects) and an “old” group (12 months old, 25 subjects). All experiments were performed according to the protocols approved by the Animal Research Center, Sun Yat-sen University.

### 2.2. Auditory Brainstem Response

Auditory brainstem response (ABR) tests were conducted using a Tucker-Davis Technology (TDT) System III (Alachua, FL, USA) as previously described [4, 5]. Briefly, mice were subcutaneously inserted with subdermal needle electrodes at the vertex (active) and below the left ear (reference) and the right ear (ground) after being anesthetized. ABR tests were measured at 8, 16, and 32 kHz. The average response to 1024 stimuli was obtained through reducing the sound intensity at 5 dB intervals near the threshold, which was defined as the lowest stimulus level where a positive wave was evident.

### 2.3. Tissue Preparation

After ABR tests, the deeply anesthetized mice were decapitated. The cochlea were obtained and fixed with 4% paraformaldehyde at 4°C overnight and decalcified in 4% sodium ethylenediaminetetraacetic acid for two days. For RNA and protein preparation, cochlear tissues were dissected and snap frozen in liquid nitrogen and stored at -80°C.

### 2.4. RNA Extraction and Sequencing

Total RNA was extracted using a TRIzol reagent (Invitrogen, USA) following the manufacturer's instructions. For RNA-sequencing, cochlear tissues from 3 mice were collected and mixed as one sample. RNA purity, concentration, and integrity were measured using a NanoPhotometer spectrophotometer (IMPLEN, USA), Qubit 2.0 Fluorometer (Life Technologies, USA), and Bioanalyzer 2100 system (Agilent Technologies, USA), respectively. LncRNA sequencing libraries were generated using a NEBNext Ultra™ Directional RNA Library Prep Kit for Illumina (NEB, USA) according to the manufacturer's recommendations. The libraries were sequenced on an Illumina Hiseq 4000 platform, and 150 bp paired-end reads were generated.

### 2.5. Differential Expression Analysis

Data filtering was performed, and clean data were obtained. The clear reads were then aligned to the reference mouse genome (GRCm38). The mapped reads of each sample were then assembled by Cufflinks (v2.1.1) in a reference-based approach. Cuffdiff (v2.1.1) was used to calculate FPKMs (expected number of Fragments Per Kilobase of transcript sequence per Million base pairs sequenced) of lncRNAs in each sample and to analyze the differential expression. Transcripts with *p*‐adjust < 0.05 were assigned as differentially expressed. Volcano plot and heat map were generated based on the differentially expressed transcripts.

### 2.6. Cell Culture

Cochlear HEI-OC1 cells (kindly provided by F. Kalinec at the House Ear Institute, Los Angeles, CA, USA) were cultured in Dulbecco's modified Eagle's medium (Gibco, USA), supplemented with 10% fetal bovine serum (Gibco) at 33°C under 10% CO_2_ condition.

### 2.7. Cell Transfection

The AW112010 Smart Silencer (Ruibo, Guangzhou, China), a commercial mixture of siRNA and ASO (antisense oligonucleotides), was used to knock down the expression of AW112010 in HEI-OC1 cell. The pcDNA3.1-AW112010 plasmid (IGE Biotechnology, Guangzhou, China) was used to express AW112010 in HEI-OC1 cell. Arid5b siRNA (GenePharma, Shanghai, China) was applied to knock down the expression of Arid5b in HEI-OC1 cell. Cell transfections were performed using a Lipofectamine RNAiMAX Transfection Reagent (Life Technologies, USA) or FuGENE® HD Transfection Reagent (Promega, USA) following the manufacturer's instructions.

### 2.8. Quantitative Reverse Transcription-Polymerase Chain Reaction (RT-qPCR)

According to the manufacturer's protocols, 500 ng total RNA was reverse-transcribed using PrimeScript RT Master Mix (Takara, Japan). A qPCR assay was performed using TB Green Premix Ex Taq II (Takara) on a Roche LightCycler 96 real-time PCR system (Roche, Switzerland). Primers for AW112010, Gm44593, 1700030C10Rik, 5330434G04Rik, Rian, and H19 were purchased from Ruibo (Guangzhou, China). Other primer sequences used were as follows: Arid5b: forward: 5′-GTGATGAGTTCGCGCCAAATC-3′, reverse: 5′-GCTGATAACTTTACCGTCACAGT-3′ and glyceraldehyde-3-phosphate dehydrogenase (GAPDH): forward: 5′-GGTCATCCATGACAACTTTGG-3′, reverse: 5′-GGCCATCACGCCACAG-3′. The expression levels of genes were normalized to GAPDH.

### 2.9. Western Blot Analysis

Western blot was conducted as previously described [4, 5]. Briefly, proteins were extracted from cells and cochlea tissues using radioimmunoprecipitation assay lysis buffer (Thermo plus, USA). Protein samples (20 *μ*g) were resolved on 10% SDS-polyacrylamide gel and transferred onto polyvinylidene fluoride membranes (Millipore, USA). After blocking with 5% nonfat milk, the membranes were incubated with anti-PGC-1*α* (1 : 1000, Abcam, USA), anti-TFAM (1 : 1000, Abcam), anti-p-AMPK (1 : 1000, Cell Signaling Technology, USA), anti-AMPK (1 : 1000, Cell Signaling Technology, USA), and anti-Arid5b (1 : 500, Abgent, USA) at 4°C overnight, followed with secondary antibodies (1 : 10000) at room temperature for 1 h. Then, the immunoreactive bands were detected using enhanced chemiluminescence (Millipore, USA). Band intensities were analyzed using NIH ImageJ. *β*-Actin was applied as loading and internal control.

### 2.10. Immunocytochemistry

After decalcification, cochlear tissues were immersed in 3% Triton X-100 solution for 30 min at room temperature. After washing with PBS, the tissues were blocked with 10% goat serum for 30 min at room temperature. Then, cochlear tissues were incubated with antibody anti-PGC-1*α* (1 : 100, Abcam) at 4°C for 24 h. After washing with PBS, cochlear tissues were incubated with Alexa Fluor 594 secondary antibody (1 : 200, Invitrogen) at 4°C overnight in darkness. Following PBS washes, the tissues were then incubated with Alexa Fluor 488 phalloidin (1 : 100, Invitrogen) at room temperature for 1 h. Then, the tissues were washed and counterstained with DAPI for 30 minutes and imaged with Zeiss LSM 710 confocal microscopy (Zeiss, Germany). Images were then analyzed using NIH ImageJ software.

### 2.11. Cell Viability Assay

Cells were treated with 1 mM H_2_O_2_ (Sigma-Aldrich, USA) for 2 h. To activate mitochondrial biogenesis, cells were preincubated with 5 *μ*M resveratrol (Sigma-Aldrich) or 0.5 *μ*M SRT1720 (Selleck, USA) for 24 h. Cell viability was detected using CCK-8 kits (DOJINDO, Japan) according to the manufacturer's protocols. Briefly, cells were plated into a 96-well plate. At the indicated time after treatments, 10 *μ*l CCK-8 solution was added into the wells. Cells were then incubated at 37°C in an incubator for 1 h. Then, a microplate reader (Labsystems Dragon, Finland) was used to measure the absorbance at 450 nm.

### 2.12. ATP Assay

An ATP assay was performed using an ATP Assay Kit (Beyotime, Shanghai, China) following the manufacturer's instructions. Briefly, cells were homogenized with lysis buffer and centrifuged at 12000 × g for 5 min at 4°C. The ATP detection reagent was diluted with dilution buffer and added into a 96-well plate. Then, samples were added into wells and mixed with detection solution. Chemiluminescence of samples and standards were measured with a SpectraMax M5 microplate reader (Molecular Devices, USA). The levels of ATP were calculated based on the standard curve and normalized to the protein content.

### 2.13. Mitochondrial Staining

Mitochondrial staining was conducted with the mitochondrial probe MitoTracker Red CMXRos (Yeasen, Shanghai, China) according to the manufacturer's protocols. Briefly, cells were incubated with CMXRos (500 nM) at 37°C in an incubator for 30 min. After washing with PBS, the cells were counterstained with DAPI for 10 minutes and imaged with an Olympus BX63 microscope (Olympus, Japan).

### 2.14. Mitochondrial Reactive Oxygen Species (ROS) Detection

Mitochondrial ROS levels were measured by staining with MitoTracker Red CM-H2XRos (Invitrogen) following the manufacturer's instructions. Briefly, cells were incubated with CM-H2XRos (500 nM) at 37°C in an incubator for 30 min. After washing with PBS, the cells were counterstained with DAPI for 10 minutes and imaged with an Olympus BX63 microscope (Olympus).

### 2.15. Mitochondrial Membrane Potential (MMP) Assay

A JC-1 Kit (GeneCopoeia, USA) was used to measure mitochondrial membrane potential following the manufacturer's instructions. Briefly, cells were incubated with a diluted JC-1 reagent (1 : 100) at 37°C in an incubator for 30 min. Then, cells were analyzed by flow cytometry with FACSVerse (BD Biosciences, USA).

### 2.16. Mitochondrial DNA (mtDNA) Content Analysis

Total genomic DNA was extracted from cells using a Universal Genomic DNA Extraction Kit (Takara) according to the manufacturer's protocols. The mtDNA levels were quantified by qPCR on a Roche LightCycler 96 (Roche) using D-loop primers (forward: 5′-GGTTCTTACTTCAGGGCCATCA-3′, reverse: 5′-GATTAGACCCGTTACCATCGAGAT-3′). Nuclear gene beta2-microglobulin (B2M) primers (forward: 5′-ATGGGAAGCCGAACATACTG-3′, reverse: 5′-CAGTCTCAGTGGGGGTGAAT-3′) were used as a nuclear control.

### 2.17. Statistical Analysis

All experiments were independently repeated at least 3 times. Data were presented as the mean ± SEM and analyzed with SPSS software and GraphPad Prism 5 software. Student's *t*-test and one-way ANOVA were used for statistical analysis. Values of *p* < 0.05 were considered significant.

## 3. Results

### 3.1. Progressive Hearing Loss with Aging in C57BL/6 Mice

**Figure 1:**
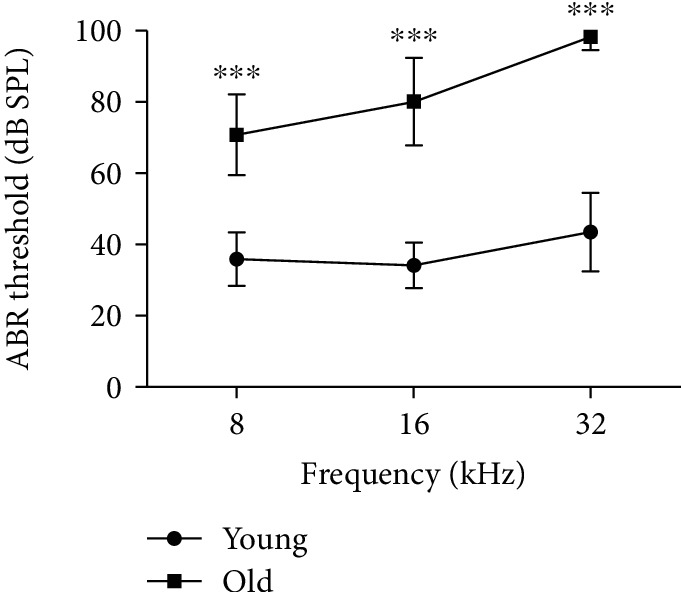
Elevated auditory brainstem response thresholds in aging C57BL/6 mice. Increased auditory brainstem response thresholds were observed in 12-month-old C57BL/6 mice at 8, 16, and 32 kHz. ^∗∗∗^*p* < 0.001 relative to 4-week-old C57BL/6 mice, *n* = 25.

Auditory brainstem response (ABR) tests were performed to examine hearing function in C57BL/6 mice. The average thresholds from old mice (71 ± 11 dB at 8 kHz, 80 ± 13 dB at 16 kHz, and 99 ± 4 dB at 32 kHz) were significantly elevated compared with those from young mice (36 ± 7 dB at 8 kHz, 34 ± 6 dB at 16 kHz, and 43 ± 11 dB at 32 kHz) (Figure 1). These data indicate that C57BL/6 mice developed early-onset AHL.

### 3.2. Differential Expression Profile of LncRNA in the Cochlea of Aged C57BL/6 Mice

**Figure 2:**
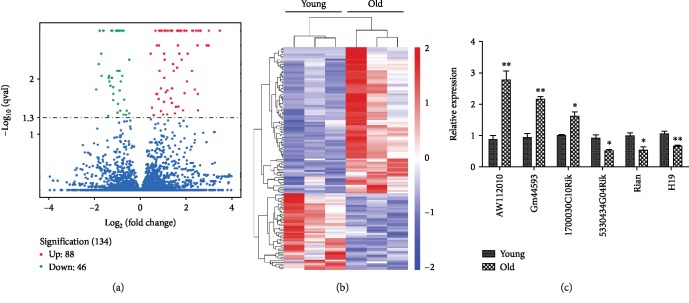
Differentially expressed lncRNAs in the cochlea of aged C57BL/6 mice. (a) Volcano plot showing comparative cochlea lncRNA gene expression in 4-week (young)- and 12-month (old)-old C57BL/6 mice (*n* = 3). Red dots indicated the significantly increased transcripts (88), and blue dots represented the significantly decreased transcripts (46) in old mice (*p*‐adjust < 0.05). (b) Heat map showing hierarchical clustering of differentially expressed lncRNAs. Red indicated high relative expression, and blue represented low relative expression. (c) RT-qPCR analysis validation of 6 differentially expressed lncRNAs. ^∗^*p* < 0.05, ^∗∗^*p* < 0.01.

To identify AHL-related lncRNAs, cochlear tissues from young and old C57BL/6 mice were collected for RNA-sequencing. Transcriptome analysis identified 88 upregulated and 46 downregulated lncRNAs in old mice compared to the young mice (*p*‐adjust < 0.05, Figure 2(a)). Volcano plot (Figure 2(a)) and heat map (Figure 2(b)) were generated using the differentially expressed lncRNAs. To confirm the sequencing results, three upregulated and three downregulated lncRNAs were randomly selected for validation using RT-qPCR. The expression patterns of all transcripts were consistent with the RNA-sequencing analysis (Figure 2(c)). These results suggest that lncRNA disorder was accompanied with the development of AHL in C57BL/6 mice.

### 3.3. AW112010 Promotes Mitochondrial Function and Cell Survival in HEI-OC1 Cells

**Figure 3:**
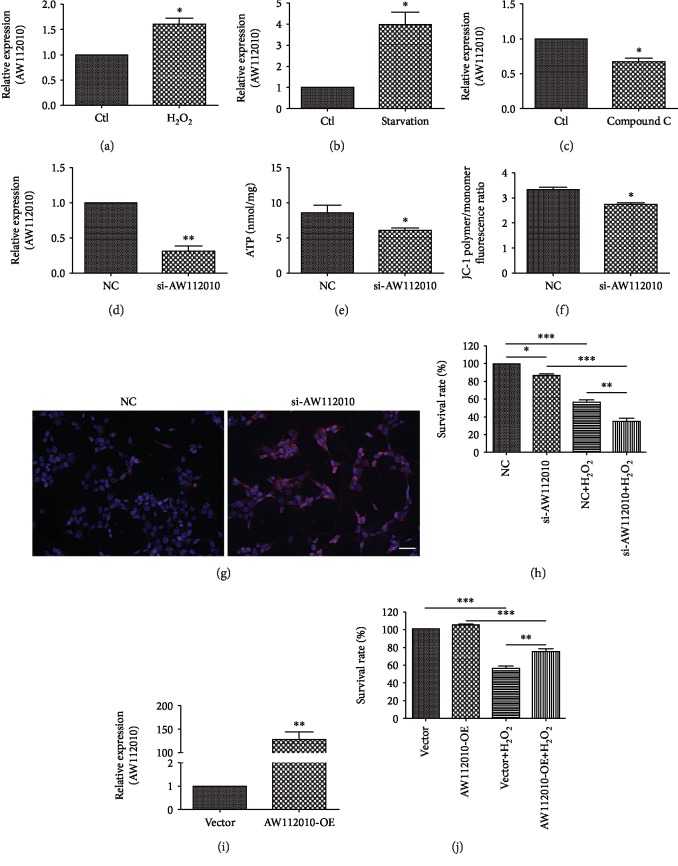
LncRNA AW112010 promoted mitochondrial function and cell survival in HEI-OC1 cells. (a) HEI-OC1 cells were treated with 1 mM H_2_O_2_ for 6 h. RT-qPCR analysis showed that H_2_O_2_ treatment increased AW112010 expression in HEI-OC1 cells. (b) Serum deprivation elevated AW112010 expression in HEI-OC1 cells. (c) HEI-OC1 cells were incubated with 5 *μ*M Compound C for 24 h. RT-qPCR analysis showed that Compound C decreased AW112010 expression in HEI-OC1 cells. (d) RT-qPCR analysis validation of AW112010 knockdown (si-AW112010) by Smart Silencer in HEI-OC1 cells. (e) ATP assay showed reduced ATP levels in AW112010 knockdown cells compared to negative control cells. (f) JC-1 assay revealed a decreased JC-1 polymer/monomer fluorescence ratio in AW112010 knockdown cells. (g) Mitochondrial ROS was measured using CM-H2XRos probes. Immunocytochemical analysis showed elevated red fluorescence in AW112010 knockdown cells under 1 mM H_2_O_2_ stimulation. Scale bar: 50 *μ*m. (h) CCK-8 assay revealed decreased cell viability in AW112010 knockdown cells in the presence or absence of 1 mM H_2_O_2_ treatment compared to negative control cells. (i) RT-qPCR analysis validation of AW112010 overexpression (AW112010-OE) in HEI-OC1 cells. (j) CCK-8 assay showed increased cell viability in AW112010 overexpression cells under H_2_O_2_ treatment. ^∗^*p* < 0.05, ^∗∗^*p* < 0.01, and ^∗∗∗^*p* < 0.001. Ctl: control; NC: negative control.

Among these dysregulated lncRNAs, we focused on the significantly upregulated gene AW112010, which has been reported to be induced following virus infection in microglia and astrocytes [14]. Firstly, we examined the expression pattern of AW112010 in HEI-OC1 cells under H_2_O_2_ treatment and serum deprivation to mimic the condition of oxidative stress and energy shortage *in vivo*. As observed in the aged cochlea sample, the expression of AW112010 was markedly induced in response to H_2_O_2_ (1 mM) stimulation (*p* < 0.05, Figure 3(a)) and serum deprivation (*p* < 0.05, Figure 3(b)). Further, Compound C, a specific AMPK inhibitor, was used to block the energy-sensitive AMPK signaling. The RT-qPCR assay showed that Compound C decreased the expression of AW112010 (*p* < 0.05, Figure 3(c)). These results indicate that AW112010 may be involved in oxidative stress and energy metabolism.

Given that the mitochondrion is the central organelle in regulating energy metabolism and oxidative stress, we wondered about the role of AW112010 in mitochondrial function. First, the expression of AW112010 was knocked down by transfection of Smart Silencer (a commercial mixture of siRNA and ASO) in HEI-OC1 cells. The knockdown efficiency was confirmed by RT-qPCR (*p* < 0.01, Figure 3(d)). Then, we measured the ATP level, mitochondrial ROS generation, mitochondrial membrane potential (MMP), and cell viability in HEI-OC1 cells. AW112010 knockdown decreased the cellular ATP level (*p* < 0.05, Figure 3(e)). The JC-1 assay showed that AW112010 inhibition decreased MMP (*p* < 0.05, Figure 3(f)). AW112010 inhibition elevated mitochondrial ROS generation under H_2_O_2_ stimulation (Figure 3(g)). The CCK-8 assay revealed that AW112010 knockdown slightly decreased cell survival (*p* < 0.05, Figure 3(h)). Under H_2_O_2_ (1 mM) stimulation, AW112010 knockdown significantly reduced the cell survival rate compared to the control group (*p* < 0.01, Figure 3(h)). Furthermore, HEI-OC1 cells were transfected with pcDNA3.1-AW112010 plasmid to express AW112010 (*p* < 0.01, Figure 3(i)). AW112010 overexpression increased cell survival in H_2_O_2_-stressed HEI-OC1 cells (Figure 3(j)). These results suggest that AW112010 promoted mitochondrial function and cell survival in HEI-OC1 cells.

### 3.4. AW112010 Induces Mitochondrial Biogenesis Process in HEI-OC1 Cells

**Figure 4:**
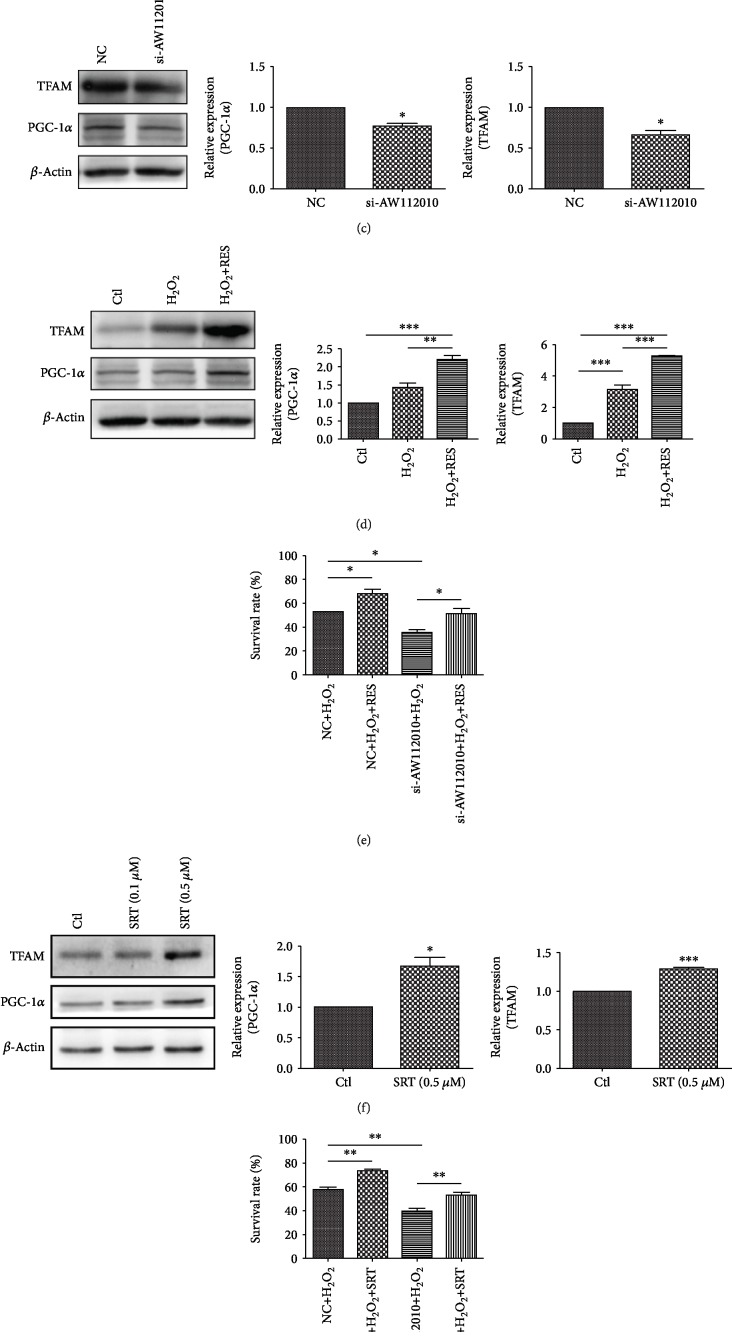
AW112010 induced mitochondrial biogenesis in HEI-OC1 cells. (a) Mitochondrial mass was measured using MitoTracker Red CMXRos probes. Mitochondrial staining showed reduced red fluorescence in AW112010 knockdown cells. Scale bar: 50 *μ*m. (b) mtDNA (D-loop) levels were detected by qPCR and normalized to nuclear gene beta2-microglobulin. Silencing of AW112010 reduced the mtDNA content in HEI-OC1 cells. (c) Western blot and densitometry of mitochondrial biogenesis-related transcription factors PGC-1*α* and TFAM. Levels of PGC-1*α* and TFAM were normalized to the *β*-actin level. AW112010 knockdown reduced the expression of PGC-1*α* and TFAM in HEI-OC1 cells. (d) HEI-OC1 cells were preincubated with resveratrol (5 *μ*M) for 24 h and then treated with H_2_O_2_ (1 mM) for 6 h. Western blot showed that resveratrol activated the expression of PGC-1*α* and TFAM in HEI-OC1 cells. (e) After AW112010 Smart Silencer transfection, cells were preincubated with resveratrol (5 *μ*M) for 24 h and then treated with H_2_O_2_ (1 mM) for 2 h. CCK-8 assay revealed that resveratrol elevated cell viability against H_2_O_2_ insults in HEI-OC1 cells. (f) HEI-OC1 cells were incubated with 0.1 or 0.5 *μ*M STR1720 for 24 h. Western blot showed that 0.5 *μ*M SRT1720 induced the expression of PGC-1*α* and TFAM in HEI-OC1 cells. (g) After AW112010 Smart Silencer transfection, cells were preincubated with SRT1720 (0.5 *μ*M) for 24 h and then treated with H_2_O_2_ (1 mM) for 2 h. CCK-8 assay revealed that SRT1720 increased cell viability against H_2_O_2_ insults in HEI-OC1 cells. ^∗^*p* < 0.05, ^∗∗^*p* < 0.01, and ^∗∗∗^*p* < 0.001. RES: resveratrol; SRT: SRT1720.

To determine whether AW112010 plays a role in mitochondrial biogenesis, the mitochondrial mass was assessed by mitochondrial probe staining. Silencing of AW112010 decreased the red fluorescence of mitochondrial staining in HEI-OC1 cells (Figure 4(a)). Further, the level of mtDNA (D-loop) was reduced in AW112010 silencing cells compared to the control (*p* < 0.05, Figure 4(b)). In addition, Western blot analysis showed that the levels of mitochondrial biogenesis transcription factors PGC-1*α* and TFAM decreased in AW112010 silencing cells (*p* < 0.05, Figure 4(c)). Resveratrol and SRT1720 have been proposed to activate mitochondrial biogenesis. Resveratrol (5 *μ*M) and SRT1720 (0.5 *μ*M) evidently increased the protein level of PGC-1*α* and TFAM in HEI-OC1 cells (Figures 4(d) and 4(f)). Treatment with resveratrol (5 *μ*M) and SRT1720 (0.5 *μ*M) protected HEI-OC1 cells against H_2_O_2_ insults (Figures 4(e) and 4(g)). These data indicate that AW112010 activated mitochondrial biogenesis in HEI-OC1 cells.

### 3.5. Activated Mitochondrial Biogenesis Process in the Cochlea of Aged C57BL/6 Mice

**Figure 5:**
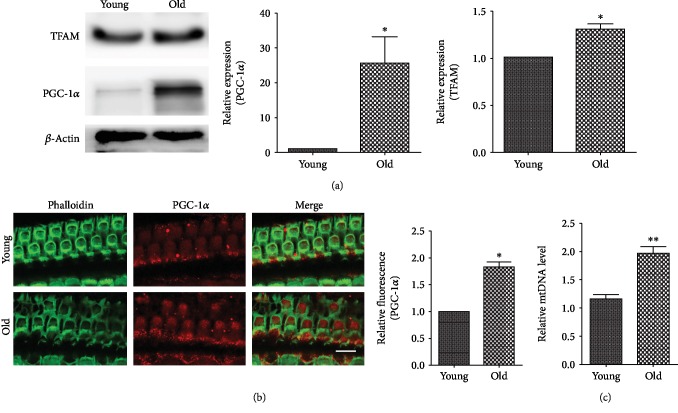
Activation of the mitochondrial biogenesis process in the cochlea of aged C57BL/6 mice. (a) Western blot and densitometry of PGC-1*α* and TFAM. Levels of PGC-1*α* and TFAM were normalized to the *β*-actin level. Increased expressions of PGC-1*α* and TFAM were detected in the cochlea of aged mice. (b) Immunocytochemical analysis showed elevated immunolabeling of PGC-1*α* in the outer hair cells of aged mice. Scale bar: 10 *μ*m. (c) qPCR analysis showed an increased mtDNA level in aged cochlea tissues. ^∗^*p* < 0.05, ^∗∗^*p* < 0.01.

To determine the status of the mitochondrial biogenesis process in the aged cochlea, cochlear tissues from young and aged C57BL/6 mice were collected for detection of mitochondrial biogenesis-related transcription factors. Western blot analysis showed that the levels of PGC-1*α* and TFAM were significantly elevated in aged cochlea samples (*p* < 0.05, Figure 5(a)). Immunocytochemical analysis further showed that the PGC-1*α* immunolabeling was increased in the outer hair cells of aged cochlea samples (*p* < 0.05, Figure 5(b)). Moreover, the mtDNA level was higher in aged cochlea tissues than in the young (*p* < 0.01, Figure 5(c)). Taken together, these findings suggest that the mitochondrial biogenesis process was activated in the cochlea of aged C57BL/6 mice.

### 3.6. AW112010 Modulates the Activation of AMPK in HEI-OC1 Cells

**Figure 6:**
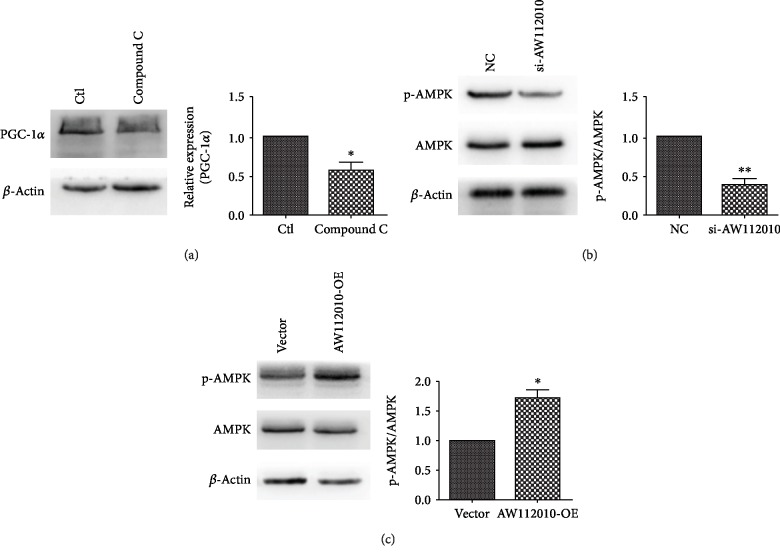
AW112010 induced the activation of AMPK in HEI-OC1 cells. (a) Western blot assay showed that AMPK inhibitor Compound C decreased the expression of PGC-1*α* in HEI-OC1 cells. (b) Western blot assay revealed that AW112010 knockdown reduced the expression of phosphorylated AMPK (p-AMPK) in HEI-OC1 cells. (c) Western blot assay showed that AW112010 overexpression increased the expression of p-AMPK in HEI-OC1 cells. ^∗^*p* < 0.05, ^∗∗^*p* < 0.01.

AMPK functions as a cellular energy sensor and is a critical mediator of mitochondrial biogenesis [11]. Inhibition of AMPK by Compound C decreased the expression of mitochondrial biogenesis transcription factor PGC-1*α* (*p* < 0.05, Figure 6(a)). To test if AW112010 may regulate mitochondrial biogenesis via the AMPK pathway, the expression of activated AMPK was detected by Western blot assays. AW112010 silencing reduced the level of phosphorylated AMPK (*p* < 0.01, Figure 6(b)), while AW112010 overexpression increased it (*p* < 0.05, Figure 6(c)). These data suggest that AW112010 positively modulated AMPK activation in HEI-OC1 cells.

### 3.7. Transcription Factor Arid5b Regulates AW112010 Expression

**Figure 7:**
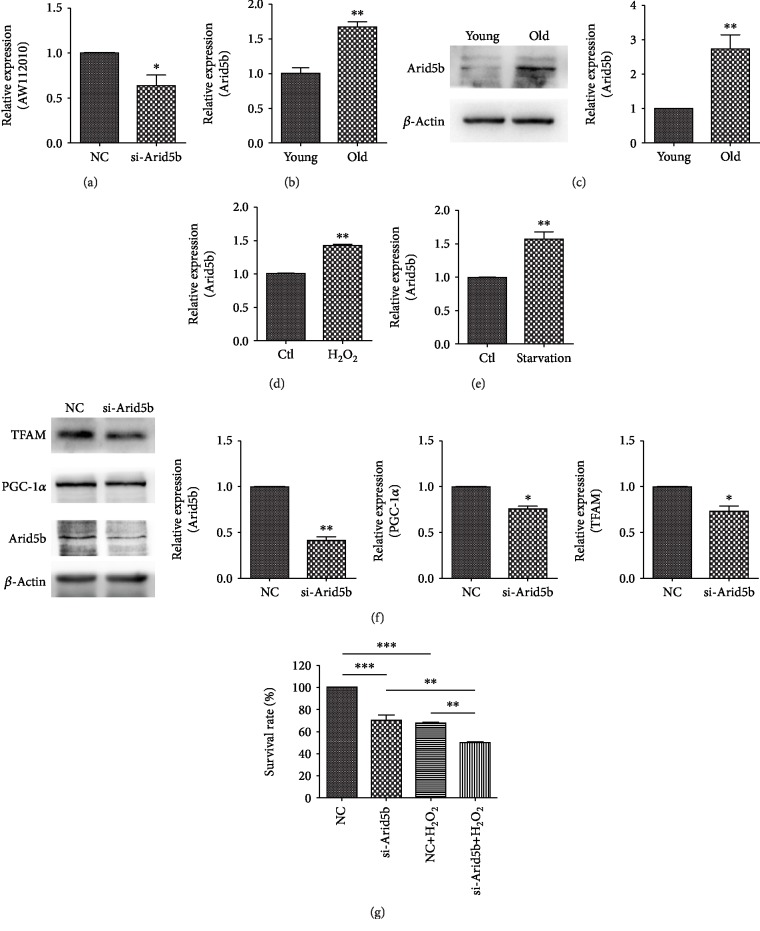
Transcription factor Arid5b modulated the expression of AW112010 and mitochondrial biogenesis process. (a) RT-qPCR assay revealed a reduced expression of AW112010 in Arid5b knockdown cells compared to negative control cells. The expression of Arid5b in the mouse cochlea was measured by RT-qPCR (b) and Western blot assay (c). Elevated mRNA and protein expression of Arid5b was detected in old mice compared to young mice. RT-qPCR assay showed an increased expression of Arid5b in HEI-OC1 cells under the treatment of H_2_O_2_ (d) and serum deprivation (e) in HEI-OC1 cells. (f) Western blot assay showed that Arid5b knockdown inhibited the expression of PGC-1*α* and TFAM in HEI-OC1 cells. (g) CCK-8 assay revealed decreased cell viability in Arid5b knockdown cells in the presence or absence of 1 mM H_2_O_2_ treatment compared to negative control cells. ^∗^*p* < 0.05, ^∗∗^*p* < 0.01, and ^∗∗∗^*p* < 0.001.

We next explored the mechanism of upregulation of AW112010 in aged cochlea samples. Using the QIAGEN database (http://www.sabiosciences.com/chipqpcrsearch.php), we found that Arid5b is the top one transcription factor for AW112010. Further, siRNA-mediated knockdown of Arid5b was performed in HEI-OC1 cells (Figure 7(f)). Arid5b knockdown cells showed a marked decrease in AW112010 expression (*p* < 0.05, Figure 7(a)). Moreover, the expression of Arid5b in the aged cochlea was examined. Similar to elevated AW112010, the Arid5b RNA and protein levels were increased in aged cochlea samples (*p* < 0.01, Figures 7(b) and 7(c)). H_2_O_2_ stimulation and serum deprivation induced Arid5b expression in HEI-OC1 cells (*p* < 0.01, Figures 7(d) and 7(e)). Additionally, Arid5b inhibition reduced the protein levels of PGC-1*α* and TFAM (*p* < 0.05, Figure 7(f)) and decreased the cell viability in HEI-OC1 cells (Figure 7(g)). These results indicate that transcription factor Arid5b positively regulated AW112010 expression and mitochondrial biogenesis.

## 4. Discussion

**Figure 8:**
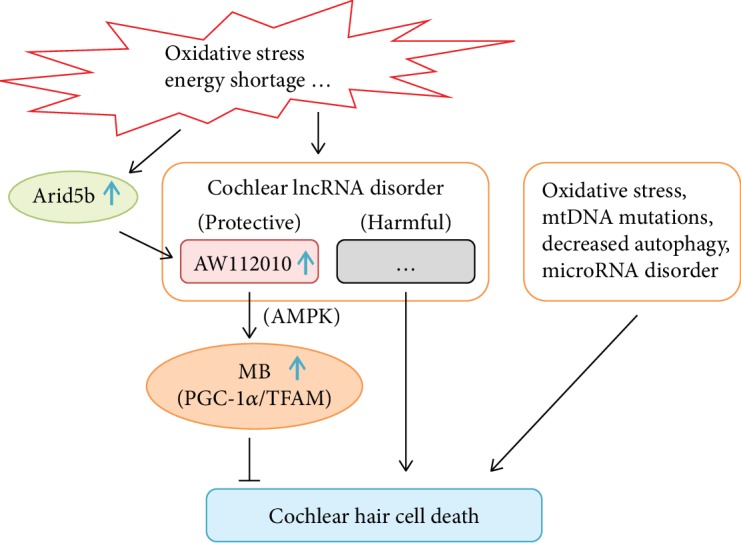
Schematic model demonstrating that activated Arid5b/AW112010 signaling promotes mitochondrial biogenesis in the cochlea. Oxidative stress, mitochondrial DNA (mtDNA) mutations, decreased autophagy, and microRNA disorder account for the death of cochlear hair cells [1, 2, 4, 5]. During aging, adverse factors such as oxidative stress and energy shortage result in the dysregulation of cochlear lncRNAs, which might have protective or harmful roles in cochlear cell survival. In these adverse conditions, transcription factor Arid5b is elevated and activates the expression of AW112010. AW112010, probably via the activation of AMPK, increases the expression of PGC-1*α* and TFAM and promotes mitochondrial biogenesis (MB) in the purpose of protecting the cochlear hair cell from death.

In this study, we identified dysregulated lncRNAs in the cochlea of aged C57BL/6 mice, which displayed early-onset AHL. We focused on the significantly upregulated AW112010 and its role in mitochondrial biogenesis. In HEI-OC1 cells, AW112010 promoted mitochondrial function and mitochondrial biogenesis. In accordance with elevated AW112010 expression, the mitochondrial biogenesis process was activated in the aged cochlea. Moreover, AW112010 positively regulated the energy-sensitive AMPK signaling in HEI-OC1 cells. In addition, transcription factor Arid5b elevated in the aged cochlea and positively modulated the AW112010 level and mitochondrial biogenesis in HEI-OC1 cells (Figure 8).

Increasing evidence has demonstrated that lncRNAs play critical roles in aging-associated diseases [8, 15–17]. In this study, to the best of our knowledge, we first revealed the lncRNA profile in the aged cochlea in an AHL mouse model. These dysregulated lncRNAs may have favorable or harmful effects on the survival of cochlear hair cells. Among these altered lncRNAs, AW112010 was significantly increased in the aged cochlea. It has been reported to be induced following virus infection in microglia and astrocytes [14], but its function has not been characterized. Cells usually suffer from excessive oxidative stress and energy shortage with aging [18]. We speculated that AW112010 might be induced under such adverse situations *in vitro*. As expected, H_2_O_2_ stimulation and serum deprivation significantly triggered AW112010 expression in HEI-OC1 cells. In addition, AMPK inhibitor Compound C markedly reduced AW112010 expression, suggesting that AW112010 is sensitive to AMPK signaling which plays a key role in energy metabolism. These findings suggest that AW112010 may be involved in oxidative stress and energy metabolism.

Mitochondria are the cellular center for energy production as well as the major source of ROS [19, 20]. Mitochondrial dysfunction is an important mechanism underlying AHL development [1]. In this study, we revealed that AW112010 has a protective role in maintaining mitochondrial function in HEI-OC1 cells. Elevated AW112010 in the aged cochlea was probably a result of adaptive mechanism to cope with the shortage of energy and insult of ROS. A progressive increase in mitochondrial mass was observed during aging in multiple cell types of diverse organisms [11, 21]. In aged tissues, cells usually suffer from energy shortage and excessive ROS. Mitochondrial biogenesis takes place under basal condition and is an adaptive response induced by cells to maintain energy demands [22]. In the present study, we observed an activated expression of PGC-1*α* and TFAM in aged mouse cochlea. PGC-1*α* is a core regulator of mitochondrial biogenesis [23, 24]. A recent study revealed that lncRNA Tug1 regulated mitochondrial bioenergetics through transcriptionally activating PGC-1*α* expression [13]. In this study, we demonstrated that AW112010 positively regulates mitochondrial mass and the expression of PGC-1*α* and TFAM in HEI-OC1 cells. It is reasonable to speculate that an elevated AW112010 level in the aged cochlea must, at least partially, account for the activated mitochondrial biogenesis process in the purpose of maintaining mitochondrial function. Resveratrol and SRT1720 have been demonstrated to promote mitochondrial biogenesis via activation of sirtuin 1/PGC-1*α* signaling [25, 26]. In our study, resveratrol and SRT1720 induced the expression of PGC-1*α* and TFAM and rescued cell viability under oxidative stress in AW112010 silencing cells. Activation of PGC-1*α* by genetic or pharmacological methods rescued the phenotypes of genetic models of Parkinson's disease [27]. Our study and others' together supported the notion that promotion of mitochondrial biogenesis may serve as an effective method to treating aging-related diseases.

Mitochondrial biogenesis is controlled by multiple signaling pathways like AMPK, which is the major energy sensor in cells [11, 28]. AMPK phosphorylates PGC-1*α* and induces mitochondrial biogenesis [11]. In this study, we observed that AW112010 induced the activation of AMPK. AW112010 might regulate mitochondrial biogenesis through AMPK signaling. To explore the upstream regulator of AW112010, we found that transcription factor Arid5b mediated the expression of AW112010 in HEI-OC1 cells. In addition, similar to the AW112010 expression pattern, the level of Arid5b was elevated in aged cochlea samples. Arid5b might be partially responsible for the overexpression of AW112010 in the aged cochlea. In addition, Arid5b positively modulated the expression of PGC-1*α* and TFAM. Under oxidative stress and energy shortage conditions, the Arid5b/AW112010 signaling might be activated and attempted to recover cellular homeostasis in the cochlea of aged mice. Arid5b has also been proposed to be an important mediator in lipid metabolism via inducing the expression of CCAAT/enhancer-binding protein *α* (C/EBP*α*) and peroxisome proliferator-activated receptor-*γ* (PPAR*γ*) in adipocytes [29, 30].

In sum, our findings, for the first time, demonstrated that lncRNAs were dysregulated with aging in the cochlea of C57BL/6 mice. The Arid5b/AW112010 signaling was activated in aged mouse cochlea and positively modulated the mitochondrial biogenesis process to maintain mitochondrial function. Our results increase our understanding of lncRNA-mediated mitochondrial biogenesis in maintaining hair cell homeostasis and provide potential targets for developing lncRNA-based therapeutics in AHL.

## Data Availability

The sequencing data have been submitted to the NCBI Gene Expression Omnibus (GEO) under accession number GSE127204.
